# Study on Hydroxychloroquinine Sulfate Being Given to the Admitted COVID -19 Positive Patients at Institute of JLNMCH, Bhagalpur, Bihar, India

**DOI:** 10.7759/cureus.26388

**Published:** 2022-06-28

**Authors:** Rajkamal Choudhary, Obaid Ali, Braj Kishore Singh

**Affiliations:** 1 Internal Medicine, Jawaharlal Nehru Medical College and Hospital, Bhagalpur, IND; 2 Internal Medicine, Uttama Medicare and Research Centre, Patna, IND

**Keywords:** india, bihar, j.l.n.m.c.h, hydroxychloroquinine sulfate, covid-19

## Abstract

Background

As the global death toll from new coronavirus illness (COVID-19) rises, the scientific community and healthcare systems are under massive pressure to manage the outbreak and develop effective medical remedies. Meanwhile, desperation has pushed practitioners, scientists, and authorities to recommend and attempt medicines with little or no proof. Despite the lack of clear and unequivocal facts supporting its efficacy and safety, hydroxychloroquine-sulfate (HCQS) has recently received substantial public and political interest in treating and prophylaxis new infectious diseases COVID-19.

Aim

To analyze the impact of HCQS in COVID-19-positive patients admitted at tertiary level government-owned Jawaharlal Nehru Medical College and Hospital (JLNMCH, Bhagalpur) Bihar.

Methods

Two hundred two RT-PCR-positive COVID-19 patients were included in this research. The study participants were randomly distributed into the intervention category and control category, each consisting of 101 study subjects. Study participants in the intervention category were administered hydroxychloroquine in 200 mg tablets. The control category was given placebo tablets that looked similar to tablets of hydroxychloroquine and were given in the same pattern. Chest X-ray PA view, 12 lead ECG, baseline hemogram including a concentration of CD4 cells, ratio between the concentration of CD8 cells and CD4 cells and concentration of CD8 cells, the concentration of C-reactive protein, RT PCR test of samples obtained from the oropharyngeal region and nasopharyngeal region nasopharyngeal for verification COVID-19 were done. These measurements were carried out in both the control and intervention categories at baseline and at the moment of obtaining a negative RT-PCR report.

Results

The confirmed cases of COVID-19 was 52.9% in the intervention category and 53.4% in the control category at the end of the administration of drugs. Symptoms related to COVID-19 were observed in 11.6% of subjects in the intervention category and 13.5% in the control category. Other new symptoms were observed in 16.8% of subjects in the intervention category and 14.5% of study subjects in the control category. One death was reported in the control category. Emergency hospitalization was required for one subject in the intervention category, while two subjects in the control category required emergency hospitalization. 12.4 days was the mean duration of recovery in the intervention category, while 13.6 days were the mean duration of recovery in the control category. The recovery duration and COVID-related symptoms were lesser in the intervention category than in the control category; however, the variation between the two categories was statistically non-significant (p˃0.01).

Conclusion

According to this research, no statistically significant difference was noticed in COVID-19 incidence between the control category and intervention category, showing that hydroxychloroquine sulfate cannot be utilized as the main curative agent in the treatment of COVID-19. However, there was a reduction in recovery days and symptoms related to COVID-19 in study subjects administered with HCQS.

## Introduction

The first case of COVID-19 was found in China. The SARS-CoV-2 virus, responsible for causing the severe acute respiratory syndrome, was believed to be the main pathogenic organism for this new coronavirus disease. It was reported for the first time during the last phase of December 2019. The WHO designated this epidemic a worldwide medical emergency by the last week of January 2020 and a pandemic by the second week of March 2020 [1].

The lung's epithelial cells are this virus's most common target. Infection begins when the virus binds to the angiotensin-converting enzyme (ACE-2) receptors, which are found on the surface of host cells. Then there is an association between the cell membrane and this virus. According to information obtained from the molecular analysis, it has been found that many cytokines and chemokines play a vital role in the establishment of COVID-19 infection [2]. The cytokines and chemokines like interferon, Granulocyte colony-stimulating factor (G-CSF), interleukin-1 cytokine, interleukin-6 cytokine, interleukin-7 cytokine, interleukin-8 cytokine, interleukin-9 cytokine, interleukin-10 cytokine, Tumor necrosis factor (TNF), etc. are involved in COVID-19 infection. The two most life-threatening complications of COVID-19 infection are failure of multiple organs and acute respiratory distress syndrome. The massive production of cytokines is responsible for the pathogenesis of two life-threatening conditions and the poor prognosis among the patients suffering from these diseases [3]. 

Rapid recognition of an infected person, confinement of the infected person, tracing of the people who came into contact with the patient, and self-isolation are the contemporary public health techniques for preventing spread. Once a person has been exposed, the standard treatment is observation and complete isolation for a fourteen-day incubation phase. No medicine has been found to restrict the communication of SARS-CoV-2 from an infected individual to another normal individual. Although the existing scientific evidence promoting their utilization seems insufficient, wide-ranging anti-viral drugs such as HIV protease inhibitors, nucleoside analogs, RNA synthesis inhibitors, and neuraminidase inhibitors, neuraminidase inhibitors could probably play a vital role in the treatment of patients affected by COVID-19. Several anti-viral drugs have been approved for emergency use, like remdesivir, favipiravir, and galidesivir, and a combination of lopinavir and ritonavir [4]. 

It has been observed during in vitro studies that chloroquine and its derivative hydroxychloroquine have been active against the SARS-CoV-2 virus and the SARS-CoV virus. Hydroxychloroquine is believed to prevent the terminal glycosylation of an enzyme's receptor, namely the ACE 2 enzyme. Because the ACE 2 receptor on the host cell's outer membrane is a site for attachment of the spike glycoprotein on the envelope of the COVID-19 virus, terminal glycosylation of the ACE inhibitor prevents the virus from binding to the host cell [5]. There is further stoppage of the function of endolysosome due to modification of the ACE 2 receptor by hydroxychloroquine. Some in vitro studies found hydroxychloroquine more effective against viruses than chloroquine. During a pandemic's early course, hydroxychloroquine sulfate was experimented with for prophylaxis and treatment of COVID-19 [6].

The scientific community believes that hydroxychloroquine-sulfate (HCQS) can be an important pharmacological option for effectively curing COVID-19-infected individuals. We do not have sufficient clinical information to support the use of HCQS in the management of COVID-19. As a result, HCQS has the potential to be an effective therapeutic option for the effective treatment of COVID-19, particularly in low and middle-income countries such as India. The mechanism of HCQS at the molecular level regarding its therapeutic action in COVID-19 has not been described adequately [7]. Some in vitro research has been performed to analyze the curative action of HCQS in COVID-19 infection. However, very few in vivo studies have been conducted to analyze the role of HCQS in curing COVID-19-infected individuals [8]. Therefore, this research was carried out to analyze the efficacy of HCQS being administered to the hospitalized COVID-19-positive individuals in mild to moderate conditions with comorbidities at Jawaharlal Nehru Medical College and Hospital (JLNMCH, Bhagalpur) in Bihar.

## Materials and methods

Study setting

It was a randomized trial where randomization was based on a computer-generated list, which was effective after recruiting the subjects in the study. The study was carried out at Jawaharlal Nehru Medical College and Hospital with IRB certificate 1695 dated 04/06/2020. Bhagalpur is a COVID-dedicated hospital covering five districts in Bihar: Bhagalpur, Madhepura, Katihar, Purnea, and Munger. As per government policy, it was designated as a referral facility for COVID-19 patients. The study was carried out between 05-06-2020 and 15-07-2020. Two hundred and two patients were kept in the Isolation Ward at Jawaharlal Nehru Medical College and Hospital, Bhagalpur. These study subjects were randomized into two categories: the intervention category, consisting of 101 topics, and the control category, consisting of 101 study subjects. Approval was obtained from the committee of the institution. Study participants were provided with an informed consent form and study literature. Every study participant was asked to sign the informed consent form before starting research work. Blinding was done one-sided where the patient was blinded (unaware of the group) and the doctor was not blinded. 

Sample size

We estimated that 10% of COVID-19-exposed close contacts would develop sickness compatible with COVID-19. We predicted that 101 people would need to be recruited in each category with the help of Fisher's exact technique, having a fifty percent relative outcome size with 90 percent power and a two-sided alpha of 0.05 as per previous research articles. Patients with confirmed (positive) COVID-19 cases (all by RT-PCR), mild to moderate clinical cases, type 2 diabetes mellitus (T2DM), hypertension (HTN), chronic kidney disease (CKD), obesity, chronic obstructive pulmonary disease (COPD), age category >= 30 years and = 60 years were eligible. Exclusion criteria excluded patients with retinopathy, hypersensitivity to HCQ or 4-aminoquinolone compounds, G6PD deficiency, pre-existing cardiac diseases, age categories 30 yr and > 60 yr, and QTc > 480 ms.

Measurements

All suspected COVID-19 patients were initially received in the hospital's isolation ward. Patients were assessed, and an entire history of COVID-19 suspects was taken, including any recent travel to elevated risky COVID-19-affected areas, a history of close contact with a laboratory-diagnosed COVID-19 individual, and a history of any comorbidities. Chest X-ray PA view, 12 lead ECG, baseline hemogram including the concentration of CD4 cells, the ratio between the concentration of CD8 cells and CD4 cells, the concentration of C-reactive protein, and RT PCR test of samples obtained from the oropharyngeal region and nasopharyngeal region for verification of COVID-19 were done. These measurements were carried out in both the control and intervention categories at baseline and when obtaining a negative RTPCR report.

Mild and moderate patients were treated in the isolation ward, whereas severe cases were transferred to the ICU of the hospital. Supplemental oxygen therapy was provided to patients. Patients have been prescribed paracetamol for fever, tablets of vitamin C, and zinc acetate. Montelukast, cetirizine, and other symptomatic treatments were given, along with treatment for other comorbidities. Azithromycin and hydroxychloroquine were prescribed to patients with respiratory symptoms.

Intervention

Study participants in the intervention category received 800 mg hydroxychloroquine stat doses followed by 600 mg 6-8 hours later. After that, they received 600 mg tablets once daily for an additional four days, and participants received 19 tablets over five days. We established this hydroxychloroquine dose regimen based on pharmacokinetic models to obtain circulating levels well above SARS-CoV-2 virus concentrations in vitro at a halfway maximum optimum amount for 14 days.

The control group was given placebo tablets that looked similar to the hydroxychloroquine tablets and were administered in the same pattern. Some pharmaceutical companies donated hydroxychloroquine, and further hydroxychloroquine was purchased. Beta-lactam was also shown in the case of pneumonia when combined with a beta-lactamase inhibitor. After five days, a follow-up RT PCR of swabs obtained from the nasopharynx and oropharynx was performed. If the test was positive, the process was followed two days later. The patient was released from the hospital after two sequential negative results.

Statistical analysis

The mean and standard deviations were used to describe quantitative data. Assessment of qualitative data was carried out with the help of percentages and frequencies. The data analysis was carried out with the help of statistical tests like independent sample t-tests, binary logistic regression, and chi-square tests. A convergence difficulty developed when we attempted to use binomial regression analysis to estimate the influence of hydroxychloroquine (HCQS) on the prevalence of COVID-19. A simplified Poisson regression method was established to calculate the values of overall RRs, i.e., risk ratios, and their 95 CIs, i.e., confidence intervals. All analyses were carried out by applying Stata software version 14. We calculated that 202 cases (101 in each category) would be needed to generate a 25% difference in COVID-19 incidence between the two types with an 80% power. 0.05 was kept as the criteria for the data to be significant statistically.

## Results

**Figure 1:**
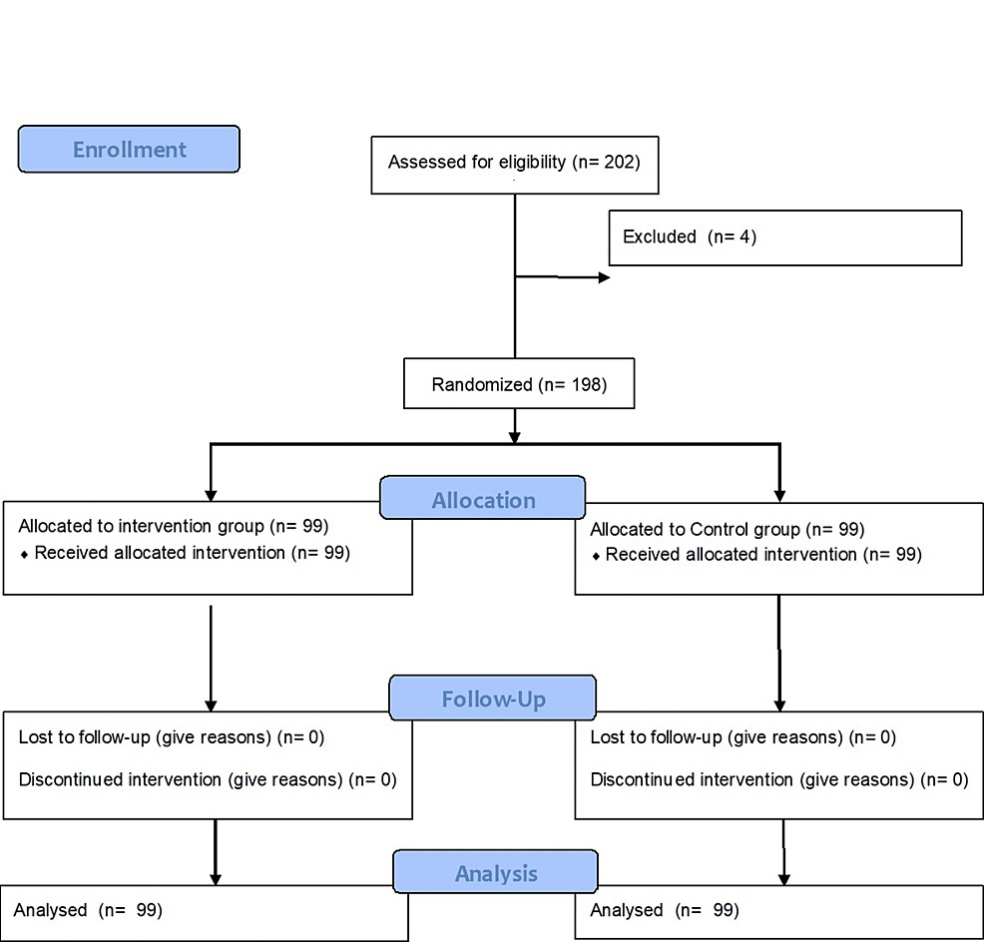
Consort flow diagram of the participants.

In this research, 101 subjects were included at the start of research in the control and intervention categories. However, two study participants in the intervention category were barred from research because of the severe adverse effects of hydroxychloroquine within 30 days. Therefore, the intervention category consisted of 99 study subjects during the trial. Similarly, two study participants in the control category were barred because they did not want to continue participating in the trial (Figure 1). Therefore control category also consisted of 99 study participants finally. The demographic details are represented in table 1.

**Table 1:** Demographic properties and clinical features of the participants at the beginning of the study.

Properties	Hydroxychloroquine category (n=99)	Control Category (n=99)	p value
Mean age	43.4 years	42.6 years	0.071
Median weight	73.2 kg	74.8 kg	0.023
Female sex	52.4 %	51.7%	0.042
Smoking	3.4 %	3.3 %	0.761
Health care worker	26.6	27.5	0.654
High-risk exposure (%)	88.4	87.3	0.765
COVID-19-related symptoms present	24.23%	23.46%	0.542

The mean age of study participants in the intervention and control categories was 43.4 and 42.6 years, respectively. The variation was non-significant statistically. (p ˃ 0.05). The mean weight of study subjects was 73.2 kg and 74.8 kg in the intervention and control categories, respectively. 52.4% of study participants in the intervention category and 51.7% in the control category were females. It was observed that 3.4% of the total individuals in the intervention category and 3.3% of the control category were smokers. The variation was statistically non-significant. (p˃0.005). 26.6% of study participants in the intervention category and 27.5% of the control category were health professionals. The percentage of high-risk exposure in the intervention category was 88.4%, and 87.3% in the control category. 24.43% of subjects in the intervention category and 23.46% of subjects in the control category had symptoms related to COVID-19 like fever, cough, and chills. The variation in the control category and intervention category was non-significant statistically. (p˃0.005).

There was an analysis regarding the number of days between the appearance of symptoms and the confirmation of COVID-19 by RT-PCR. It was observed that this duration was one day in 18.5% of study participants in the intervention category and 15.7% in the control category. The duration was two days for 24.6 % and 26.2% of study participants in the intervention and control categories, respectively. 23.8% and 24.2% of study participants in the intervention category and control category, respectively, were found to have a duration of 3 days, while 31.8% and 31.3% of study subjects in the intervention category and control category had a gap of four days. The variation between the control category and the intervention category was non-significant statistically. (p˃0.05) (table 2).

**Table 2:** Time between the appearance of symptoms and getting an RT-PCR positive test report.

No of days	Hydroxychloroquine category (n=99)	Control category (n=99)	p value
1	18.4 %	15.7%	0.0651
2	24.6%	26.2%	
3	23.8%	24.2%	
4	31.8%	31.3%	

Details were recorded regarding the presence of comorbidities in study participants in the intervention and control categories. (Table 3). 53.7% and 52.8% of subjects in the intervention and control categories did not have any comorbidity. Hypertension was found in 12.4% and 11.6% of subjects in the intervention and control categories, respectively. Asthma was observed in 7.7% of the intervention category and 7.8% of the control category. Diabetes was found in 3.4% of people in the intervention group and 3.8% in the control group. In 21.2% and 20.4% of subjects, intervention and control categories were found in both hypertension and diabetes. The variation was non-significant statistically (p˃0.05) (table 3). Outcomes after the end of the intervention process were analyzed and have been represented in table 4.

**Table 3:** Comorbidities associated with COVID-19

Comorbidity	Hydroxychloroquine category (n=99)	Control category (n=99)	P value
None	53.7%	52.8%	0.021
Hypertension	12.4%	11.6%	
Asthma	7.7 %	7.8%	
Diabetes	3.4 %	3.8%	
Hypertension+ Diabetes	21.2%	20.4%	
Asthma+ Hypertension	12.23%	11.12%	
Hypertension+Asthma	10.21%	11.12%	

**Table 4:** Primary and secondary outcomes at the end of the intervention.

Outcome	Hydroxychloroquine category (n=99)	Control category (n=99)	P Value
Laboratory-confirmed diagnosis	52.9%	53.4 %	0.84
Covid-19-related symptoms	11.6%	13.5%	0.42
Other new symptoms	16.8 %	14.5%	0.81
Death	0	1	-
Any emergency hospitalization	1	2	0.71
No of days for recovery	12.4	13.6	

The percentage of cases of COVID-19 confirmed through laboratory investigation was 52.9% in the intervention category and 53.4% in the control category. Symptoms related to COVID-19 were observed in 11.6% of subjects in the intervention category and 13.5% of subjects in the control category. There was an increased reduction in symptoms in the intervention category compared to the control category; however, the variation was non-significant and statistically insignificant. ( p˃0.01) Other new symptoms were observed in 16.8% of subjects in the intervention category and 14.5% in the control category. The frequency of new symptoms was greater in the intervention category than in the control category, but the variation was not statistically significant. One death was reported in the control category. Emergency hospitalization was required for one subject in the intervention category, while two subjects in the control category required emergency hospitalization. 12.4 days was the mean duration of recovery in the intervention category, while 13.6 days was the mean duration of recovery in the control category. The recovery duration was less in the intervention category than in the control category, but the variation was not statistically significant (p˃0.01).

Table 5 shows the values of various variables such as mean WBC, mean polymorphonuclear cell count, mean lymphocyte cell count, mean platelet count, mean CD4 count, mean CD8 count, and mean ratio of CD4 cell to CD8 cell, and mean CRP at baseline. The variation between the intervention category and control category is non significant in mean WBC ( p=0.92), , mean polymorphonuclear cell count ( p=0.35), mean lymphocyte cell count (p=0.74) mean platelet count (0.85), mean CD4 ( p=0.47), mean CD8 (0.87), mean ratio between CD4 cell and CD8 cell (p=0.87). The variation in the values of C-reactive protein in both the control and intervention categories was statistically non-significant. (p = 0.33) (table 5). The mean variation in WBC, PMN, lymphocyte count, platelet count, and hemoglobin at the start and the intervention's end in the control category have been described in table 6.

**Table 5:** Comparison of values of different parameters at baseline between Hydroxycholoroquinone category and control category WBC: white blood cell; PMN: Polymorphonuclear leukocytes ; LYM: lymphocytes; Hb: hemoglobin; CD: cluster of differentiation; SD: standard deviation

Parameters	Hydroxycholoroquinine category (n= 99)	Control Category (n=99)	P value
Mean WBC concentration ±S.D values	7.9± 2.6	7.5± 2.7	0.92
Mean PMN concentration, ±S.D values	67.3 ± 9.3	66.7 ± 9.5	0.35
Mean LYM concentration ±S.D values	45.3± 7.7	46.8 ±8.8	0.74
Mean Hb concentration ± S.D values	14.1 ±2.8	12.8 ±2.7	0.67
Mean Platelet concentration ± S.D values	346. 7 ±4.7	346.4 ±67.6	0.85
Mean CD4 concentration ± S.D values	47.3 ±8.7	45.7 ±8.2	0.47
Mean CD8 concentration ± S.D values	32.5 ±6.7	34.8 ±8.3	0.87
Mean Ratio of CD4 concentration to CD8 concentration ±S.D values	2.75 ±0.45	2.63 ±0.58	0.98

**Table 6:** Mean difference between the measured variables after and before intervention *: statistically significant WBC: white blood cell; PMN: Polymorphonuclear leukocytes; LYM: lymphocytes; Hb: hemoglobin; CD: cluster of differentiation; SD: standard deviation

Variables	Mean at the time of discharge of patient			Mean Difference (After-Before)		
	Hydroxycholoroquinine category (n=99)	Control Category (n=99)	P value	Hydroxycholoroquinone category (n= 99)	Control Category (n=99)	P value
Mean WBC concentration ±S.D values	8.47 ± 2.8	8.22±2.9	0.68	0.46±2.7	0.34±2.6	0.76
Mean PMN concentration, ±S.D values	67.9 ±9.8	67.5±7.8	0.79	0.77±9.7	2.5±7.6	0.71
Mean LYM concentration ±S.D values	44.4±9.5	45.3 ± 7.8	0.52	-2.1 ± 0.3	-2.1±6.9	0.42
Mean Hb concentration ± S.D values	14.3 ±0.53	14.2±2.23	0.52	-1.32±2.3	-0.22 ±2.8	0.72
Mean Platelet concentration ± S.D values	356.2±69.3	341.6 ± 74.2	0.81	21.2 ±72.7	5.1 ±58.2	0.31
Mean CD4 concentration ± S.D values	47.4±9.9	47.6 ± 6.1	0.32	0.42±9.2	5.3±9.2	0.04^*^
Mean CD8 concentration ± S.D values	37.6±6.5	38.2±7.2	0.92	4.2 ±8.21	4.2±6.8	0.05*
Mean Ratio of CD4 concentration to CD8 concentration ±S.D values	2.5±0.4	2.6±0.1	0.62	-1.29 ±0.47	-0.13 ±0.22	0.02*

The variation was non-significant statistically. When there was an analysis of the mean concentration of CD4 cell count, mean concentration of CD8 cell count. The concentration ratio of CD4 to CD8 cells observed that a decrease in the values from baseline to the end of the intervention was more in the intervention category than the control category. The variation was statistically significant (p≤0.05).

## Discussion

The authors of this study researched the therapeutic impact of hydroxychloroquine medicine on COVID-19-infected individuals in tertiary-level hospitals. According to our data, no significant statistical variation in COVID-19 occurrence was observed between the control and intervention categories, showing that hydroxychloroquine was not an effective therapeutic agent in managing COVID-19. There was a reduction in the recovery days and symptoms related to COVID-19 in the study subjects of the intervention category. In the intervention category, there was an overall increase in new symptoms related to the adverse effects of hydroxychloroquine. The two categories had some serologic differences also. The mean CD4 in the control category grew substantially after the intervention (relative to the baseline mean), but decreased in the test category. This information is worrying because it shows that hydroxychloroquine does not wholly stop COVID-19 infection, but it may weaken the immune system by stopping CD4 cells from multiplying.

Our findings are consistent with those of a randomized controlled trial-based study conducted by Boulware and colleagues, which revealed that the HCQS drug has no curative action in COVID-19. Such findings are also consistent with the findings of two previous studies, namely a retrospective study conducted on the general population by Gendelman et al. and a prospective study conducted on individuals suffering from Systemic Lupus Erythematosus by Konig et al., both of which concluded that hydroxychloroquine had no curative action against the COVID-19 infection. The findings are appropriately timed by the WHO's supervised solidarity pharmaceutical trial; other systematic literature reviews recommend further research involving drugs that can be modified in different situations [9-11].

Our findings on hydroxychloroquine's unfavorable effects on CD4 cell production are similar to those advocated for its usage in other diseases (e.g., rheumatoid arthritis). The concentration of CD4 cells and CD8 cells did not vary between COVID-19 affected individuals compared to normal healthy people. According to other research, however, CD8 expression was much greater in COVID-19-affected individuals than in healthy subjects. Furthermore, patients with COVID-19 were shown to have strong Th1 and Th17 cytokine profiles. Surprisingly, there is data that perhaps the COVID-19 virus reduces the concentration of CD4 cells and the concentration of CD8 cells in affected people [12-14].

There are two possibilities regarding the action of HCQS in COVID-19: first, individuals who have received hydroxychloroquine will have a compromised immune system due to which HCQS becomes inactive as a prophylactic or therapeutic medication; second, there can be a lowering of CD4 cell count and an elevation of CD8 cell count after administration of hydroxychloroquine. This might provide some prevention against the severe COVID-19 conditions. It means a reduction in the number of cells involved in a sudden outburst of cytokine production, namely CD4 cells, and enhancement of body defense by promoting the production of CD8 cells. When these findings are considered together, they may give more weight to warnings about the different harmful effects of hydroxychloroquine administration. One of the most extensive and up-to-date assessments on the curative action of hydroxychloroquine recommended that its usage should be restricted to specific situations where its complications can be monitored [15-16].

There is currently a scarcity of unambiguous and persuasive clinical evidence to assure the application of hydroxychloroquine in curing individuals affected by COVID-19. Several studies have been criticized for methodological issues such as small sample sizes, vaguely defined objectives, and randomization inadequacies, among other drawbacks in study design [17]. An experimental study carried out by Gautret et al. was the first clinical study that drew the attention of doctors, scientists, patients, and health officials. They concluded that combining azithromycin with HCQS has a collective impact on SARS-CoV-2 infection as the combination reduces respiratory virus load considerably [18]. In total, 22 patients diagnosed with infections of the upper respiratory tract, eight patients diagnosed with infections of the lower respiratory tract, and six asymptomatic individuals were included in this investigation.

Interestingly, after three to six days of therapy, the study found that HCQS effectively lowered the viral load. However, two patients failed to respond to HCQS therapy in their analysis. The study piqued the interest of many parties, though a few days later, it sparked major controversy concerning its methodology and inclusion criteria, among other things. 

Thirty patients diagnosed with COVID-19 have been included in a randomized prospective pilot trial described by Chen J et al., and the safety and effectiveness of HCQS were assessed. Patients were randomly assigned to receive HCQS 400 mg once daily for five days combined with customary or customary treatment alone. On day 7, there was no significant difference between the HCQS (86.7 percent) and control category (93.3 percent) regarding the percentage of COVID-19 negative individuals according to the examination of throat samples. Furthermore, no statistical variation was observed between the HCQS category and control category regarding the median time from the moment of admission to the hospital to the moment of achieving a negative report for COVID-19 infection. Temperature normalization and radiological advancement were also comparable. Researchers showed no significant advantage of utilizing HCQS in COVID-19-affected individuals, and the authors stated that larger studies are required to evaluate the usefulness of HCQS in the care of COVID-19 patients [19,20].

Because there are few studies on COVID-19, the research design and its conduction as a clinical trial are strong points of our research. Furthermore, because this study was undertaken at the start of the crisis in a single location, one of its drawbacks is the small sample size; consequently, similar studies in other communities should be conducted with bigger sample size.

## Conclusions

According to the findings of this research, no statistically significant difference was noticed in COVID-19 incidence seen between the control category and the intervention category, showing that hydroxychloroquine sulfate cannot be utilized as the main curative agent in the treatment of COVID-19. However, there was a reduction in the days of recovery and symptoms related to COVID-19 in study subjects administered with HCQS.
